# Physiological benefits evaluated by quantitative flow ratio in patients with reduced left ventricular ejection fraction who underwent percutaneous coronary intervention

**DOI:** 10.1186/s12872-020-01814-5

**Published:** 2020-12-14

**Authors:** Jiaxin Zhong, Qin Chen, Long Chen, Zhen Ye, Huang Chen, Jianmin Sun, Juchang Hong, Mingfang Ye, Yuanming Yan, Lianglong Chen, Yukun Luo

**Affiliations:** 1 Department of Cardiology, Fujian Medical University Union Hospital, No. 29 Xin Quan Road, Fuzhou, 350001 Fujian People’s Republic of China; 2 Fujian Institute of Coronary Artery Disease, Fuzhou, 350001 Fujian People’s Republic of China; 3 Fujian Heart Medical Center, Fuzhou, 350001 Fujian People’s Republic of China

**Keywords:** Quantitative flow ratio, Left ventricular ejection fraction, Percutaneous coronary intervention, Fractional flow reserve, Flow physiology

## Abstract

**Background:**

To explore the clinical benefits of revascularization in patients with different levels of left ventricular ejection fraction (LVEF) from the perspective of quantitative flow ratio (QFR).

**Methods:**

Patients who underwent successful percutaneous coronary intervention (PCI) and one-year angiographic follow-up were retrospectively screened and computed by QFR analysis. Based on their LVEF, 301 eligible patients were classified into reduced LVEF (≤ 50%, n = 48) and normal LVEF (> 50%, n = 253) groups. Pre-PCI QFR, post-PCI QFR, follow-up QFR, late lumen loss (LLL), LVEF and major adverse cardiovascular and cerebrovascular events (MACCEs) at one year were compared between groups.

**Results:**

The reduced LVEF group had a lower mean pre-PCI QFR than the normal LVEF group (0.67 ± 0.16 vs. 0.73 ± 0.15, *p* = 0.004), but no significant difference was found in the post-PCI or one-year follow-up QFR. No association was found between LVEF and QFR at pre-PCI or follow-up. The reduced LVEF group had greater increases in QFR (0.27 ± 0.18 vs. 0.22 ± 0.15, *p* = 0.043) and LVEF (6.05 ± 9.45% vs. − 0.37 ± 8.11%, *p* < 0.001) than the normal LVEF group. The LLL results showed no difference between the two groups, indicating a similar degree of restenosis. The reduced LVEF group had a higher incidence of MACCEs (14.6% vs. 4.3%, *p* = 0.016), which was mainly due to the higher risk of heart failure (6.3% vs. 0%, *p* = 0.004).

**Conclusion:**

Compared to the corresponding normal LVEF patients, patients with reduced LVEF who underwent successful PCI were reported to have greater increases in QFR and LVEF, a similar degree of restenosis, and a higher incidence of MACCEs due to a higher risk of heart failure. It seems that patients with reduced LVEF gain more coronary benefits from successful revascularization from the perspective of flow physiology evaluations.

## Background

The frequency of percutaneous coronary intervention (PCI) in patients diagnosed with coronary artery disease (CAD) is significantly increasing due to the evolution of drug eluting stents (DESs) and other novel angioplasty technologies. For patients with multivessel lesions or complex lesions, the performance of PCI treatment has been significantly improved [1]. However, considering the impact of myocardial ischaemia, myocardial stunning, myocardial hibernation and the presence of scars, patients with reduced left ventricular ejection fraction (LVEF) may be likely to develop complicated conditions. Moreover, the consideration of long-term benefits still makes it controversial whether such patients should undergo revascularization in clinical practice. Therefore, rational evaluation before intervention and during follow-up is of particular importance for PCI patients [2].

Fractional flow reserve (FFR) has been widely recognized as the gold standard for the assessment of coronary ischaemia requiring revascularization [3]. A large number of studies, such as the FAME study or the FAME II study, have confirmed that FFR-guided PCI can lead to more long-term benefits than traditional angiography-guided PCI [4–6]. Coronary functional assessment plays an important role in the overall assessment of CAD patients. Nonetheless, FFR assessments are still largely under-utilized in clinical practice due to concerns regarding prolonged procedural time, increased costs and potential complications caused by pressure-wire instrumentation [7].

Quantitative flow ratio (QFR) is an up-and-coming angiography-based approach that allows for the fast computation of FFR by three-dimensional (3D) coronary artery reconstruction and fluid dynamics computation [7]. The FAVOR pilot study confirmed that the accuracy of QFR on ischaemic evaluation, with FFR as a reference, was as high as 86% [8]. Subsequent studies, such as the FAVOR II study and the FAVOR III study, further verified the accuracy of QFR in the diagnosis and evaluation of coronary ischaemia [7, 9, 10]. Furthermore, no requirement for pressure wires and reduced procedural time make QFR a suitable choice for the evaluation of coronary ischaemia [10].

Although previous studies have demonstrated that QFR is an important and reasonable component in the assessment of CAD patients, the specific application and evaluation value of QFR in CAD patients with reduced LVEF still lacks relevant research support. In addition, the potential relationships between QFR and LVEF also lack evidence. Therefore, our study aims to explore the value of QFR in pre-PCI and follow-up evaluations by comparing the pre-PCI and follow-up characteristics of patients with different LVEFs who underwent PCI.

## Materials and methods

### Study design

From April 2015 to June 2016, all consecutive patients who underwent successful PCI and one-year angiographic follow-up at Fujian Medical University Union Hospital were recruited. All enrolled patients underwent retrospective computations for QFR, and the clinical characteristics of their pre-PCI, post-PCI and one-year angiographic follow-up data were collected. Post-PCI indicates the immediate time after successful PCI. The patients were divided into the reduced LVEF group (rLVEF, LVEF ≤ 50%) and normal LVEF group (nLVEF, LVEF > 50%) according to a threshold of 50% of the pre-PCI LVEF value. This study was approved by the Ethics Committee of Union Hospital, Fujian Medical University (No. 2020KY098).

### Patient population

The study population is composed of adults who underwent coronary angiography with PCI and DES implantation. Patients diagnosed with stable angina, unstable angina, or post-acute myocardial infarction (≥ 72 h) were eligible for enrolment when the angiographic inclusion criteria were met. In addition, the indications for QFR computation were as follows: (1) diameter stenosis (DS) between 50–90% (visual assessment) due to at least one lesion, and (2) reference vessel diameter size ≥ 2.5 mm (visual assessment). Patients with any of the following clinical characteristics were excluded: (1) acute myocardial infarction (AMI) within 72 h; (2) lack of angiographic follow-up; and (3) unavailable LVEF data. Patients were further excluded if the QFR computation was no possible: (1) only one lesion with DS% > 90% and Thrombolysis in myocardial infarction (TIMI) grade < 3; (2) interrogated lesion at the site of a myocardial bridge; (3) interrogated lesion in a bypass graft; (4) poor angiographic image quality; and (5) severe overlap in the stenosed segment or severe tortuosity of any interrogated vessel.

### Data collection

The following parameters were retrospectively collected using medical records: age, sex, smoking history, AMI or PCI history, and clinical comorbidities including hypertension, diabetes, prediabetes, and renal insufficiency. Serum biochemical indexes such as glucose, low-density lipoprotein (LDL), N-terminal pro brain natriuretic peptide (NT-proBNP), C-reactive protein (CRP), and troponin I were measured in the hospital clinical laboratory using routine automated techniques.

### QFR computation

The QFR computations in our study were performed by the AngioPlus system (Pulse Medical Imaging Technology Shanghai, China) according to standard operating procedures. Two angiographic images with a minimal 25° separation in projection angles were transferred to the AngioPlus system. QFR computation was based on the contrast flow model, which incorporates contrast flow velocity in accordance with modified TIMI frame counts. Three-dimensional (3D) reconstruction of the vessel provided anatomical information of the target vessel, comprising the lumen diameter and lesion length. The operators used this information to choose an appropriate calculation approach. An end-diastolic frame was selected for each projection, and the images preferably had frames from the same cardiac cycle. The reference vessel was constructed by fitting it to healthy segments ideally proximal and distal to the lesion of interest. In addition, 3D reconstruction of the vessel provided quantitative coronary angiography information of the target vessel, including minimal lumen diameter (MLD) and late lumen loss (LLL). LLL was defined as the difference between post-PCI and follow-up MLD and is recognized as a parameter reflecting the level of vessel restenosis. The QFR computations were performed by researchers blinded to the grouping results based on LVEF.

### Clinical follow-up

The relevant clinical data and major adverse cardiovascular and cerebrovascular events (MACCEs) of all enrolled during their hospitalization were recorded. A MACCE was defined as the composite of any myocardial infarction (MI), stroke, rehospitalization related to heart failure, or any ischaemia-driven repeat revascularization. All patients were treated according to the clinical guidelines recommended at the time of discharge. The occurrence of MACCEs within one year was recorded by telephone follow-up and from reviewing medical records.

### Statistical analysis

Categorical variables are presented as counts and percentages; continuous variables are reported as the mean and standard deviation, and if normally distributed, these variables are reported as medians and interquartile range. Normality was tested with the Kolmogorov–Smirnov test or Shapiro–Wilk test appropriately. Comparisons between continuous variables were evaluated with Student’s *t* test, Welch’s *t* test, or Mann–Whitney U test depending on the results of the Levene test and normality test. Comparisons between categorical variables were performed with Pearson’s χ^2^ test or Fisher’s exact test, as appropriate. Spearman's correlation analysis was used to evaluate the relationship between two variables. A two-sided *p* value < 0.05 was considered statistically significant. All analyses were performed with SPSS 26.0 (IBM Inc., New York, NY, USA) and Prism GraphPad 8.0 (GraphPad Software Inc., La Jolla, CA, USA).

## Results

**Fig. 1:**
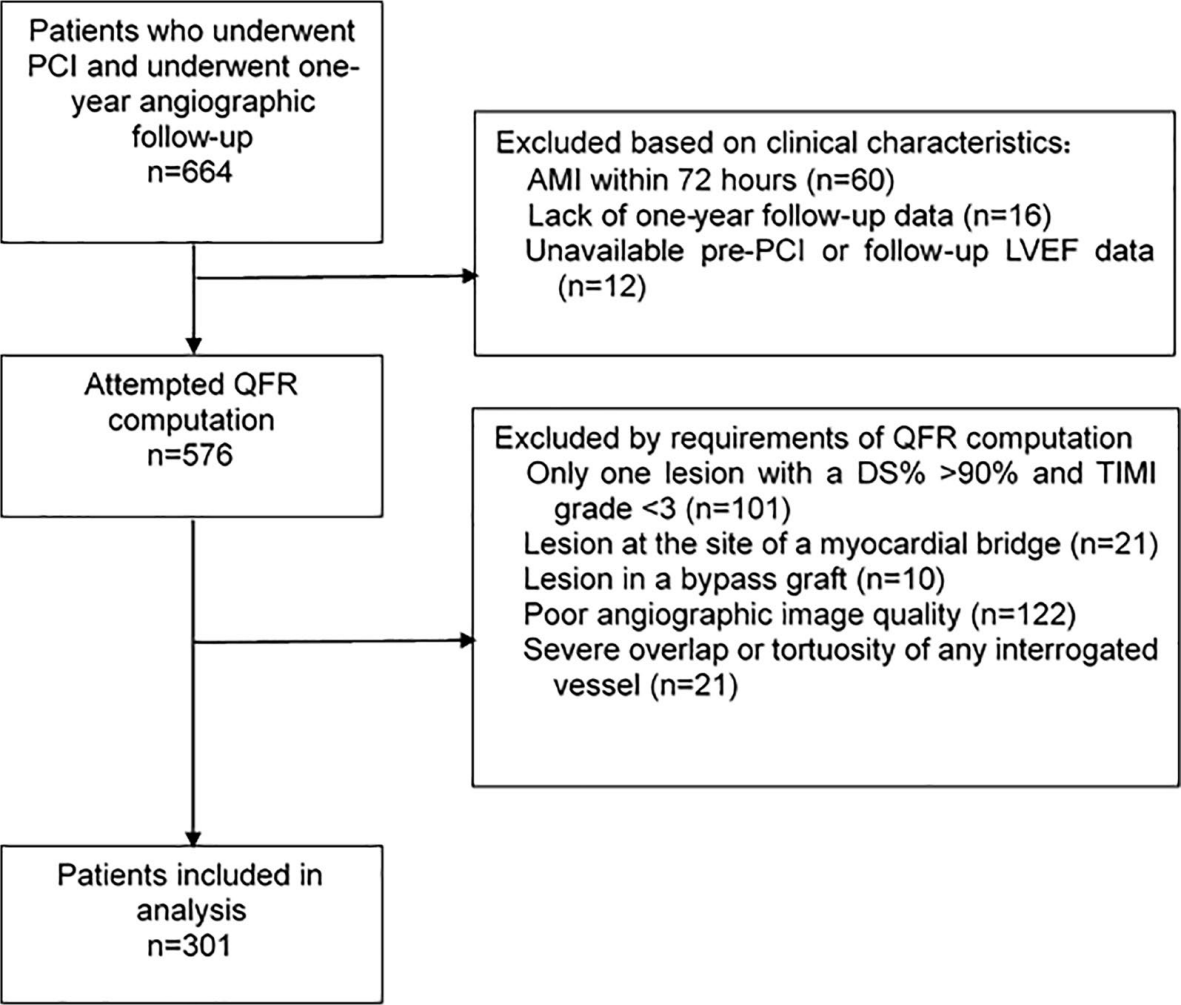
Flow chart. PCI, percutaneous coronary intervention; AMI, acute myocardial infarction; LVEF, left ventricular ejection fraction; QFR, quantitative flow ratio; DS%, diameter stenosis percentage; TIMI, thrombolysis in myocardial infarction

This study included a total of 664 patients. After excluding patients based on the predefined criteria (Fig. 1), 301 patients were enrolled in the final analysis and were divided into a reduced LVEF (rLVEF) group (n = 48) and a normal LVEF (nLVEF) group (n = 253) according to a threshold of 50% of the pre-PCI LVEF value (Fig. 1).

### Baseline characteristics

Comparisons of clinical, laboratory, and angiographic characteristics between the reduced and normal LVEF groups are summarized in Table 1. There were no differences between the two groups in age, sex, hypertension, renal insufficiency, hyperlipidaemia, smoking, or history of previous AMI or PCI. The rLVEF group had a higher incidence of diabetes (50.0% vs. 30.4%, *p* = 0.008) and a similar incidence of prediabetes. Moreover, the glucose level at admission was higher in the rLVEF group (6.70 (3.14) vs. 5.54 (1.64), *p* = 0.001). Higher levels of NT-proBNP, CRP and troponin I were recorded in the rLVEF group (all *p* < 0.05). The incidence of NSTEMI (non-ST segment elevation myocardial infarction) and STEMI (ST segment elevation myocardial infarction) was more frequent in the rLVEF group (31.3 vs. 9.9%, *p* < 0.001 and 35.4 vs. 9.9%, *p* < 0.001). The probability of stable angina pectoris was similar. Similar results in the composition of the target vessel type were found between the two groups.

**Table 1:** Baseline demographic characteristics

	Reduced LVEF group (n = 48)	Normal LVEF group (n = 253)	*P* value
*Clinical characteristics*			
Age, years	63.69 ± 8.10	63.03 ± 9.60	0.676
Male, n (%)	42 (87.5%)	197 (77.9%)	0.130
Hypertension, n (%)	31 (64.6%)	171 (67.6%)	0.684
Diabetes, n (%)	24 (50.0%)	77 (30.4%)	0.008
Prediabetes, n (%)	0 (0.00%)	7 (2.8%)	0.520
Normoglycaemic, n (%)	24 (50.0%)	169 (66.8%)	0.026
Renal insufficiency, n (%)	3 (6.3%)	9 (3.6%)	0.637
Current or previous smoker, n (%)	32 (66.7%)	139 (54.9%)	0.133
Previous myocardial infarction, n (%)	9 (18.8%)	27 (10.7%)	0.114
Previous PCI, n (%)	12 (25.0%)	51 (20.2%)	0.450
*Disease types*			
Stable angina, n (%)	2 (4.2%)	31 (12.3%)	0.100
Unstable angina, n (%)	14 (29.2%)	172 (68.0%)	< 0.001
Post-NSTEMI (≥ 72 h), n (%)	15 (31.3%)	25 (9.9%)	< 0.001
Post-STEMI (≥ 72 h), n (%)	17 (35.4%)	25 (9.9%)	< 0.001
*Laboratory indexes*			
Glucose level at admission, mmol/L	6.70 (3.14)	5.54 (1.64)	0.001
Blood LDL level at admission, mmol/L	2.92 ± 0.91	2.74 ± 0.93	0.169
NT-proBNP, pg/mL	819.50 (1412)	109.00 (220)	< 0.001
Troponin I, ug/L	5.36 ± 10.81	1.86 ± 7.93	< 0.001
CRP, mg/L	5.23 (8.94)	1.69 (4.51)	< 0.001
*Interrogated vessel*			
Left anterior descending branch, n (%)	26 (54.2%)	146 (57.7%)	0.649
Circumflex branch, n (%)	7 (14.6%)	49 (19.4%)	0.435
Right coronary artery, n (%)	15 (31.3%)	58 (22.9%)	0.217

Values are the mean ± standard deviation, median (interquartile range), or n (%)

LVEF, left ventricular ejection fraction; PCI, percutaneous coronary intervention; NSTEMI, non-ST segment elevation myocardial infarction; STEMI, ST segment elevation myocardial infarction. LDL, low density lipoprotein; NT-Pro BNP, N-terminal pro brain natriuretic peptide; CRP, C-reactive protein

### Relationship between LVEF and QFR

**Fig. 2:**
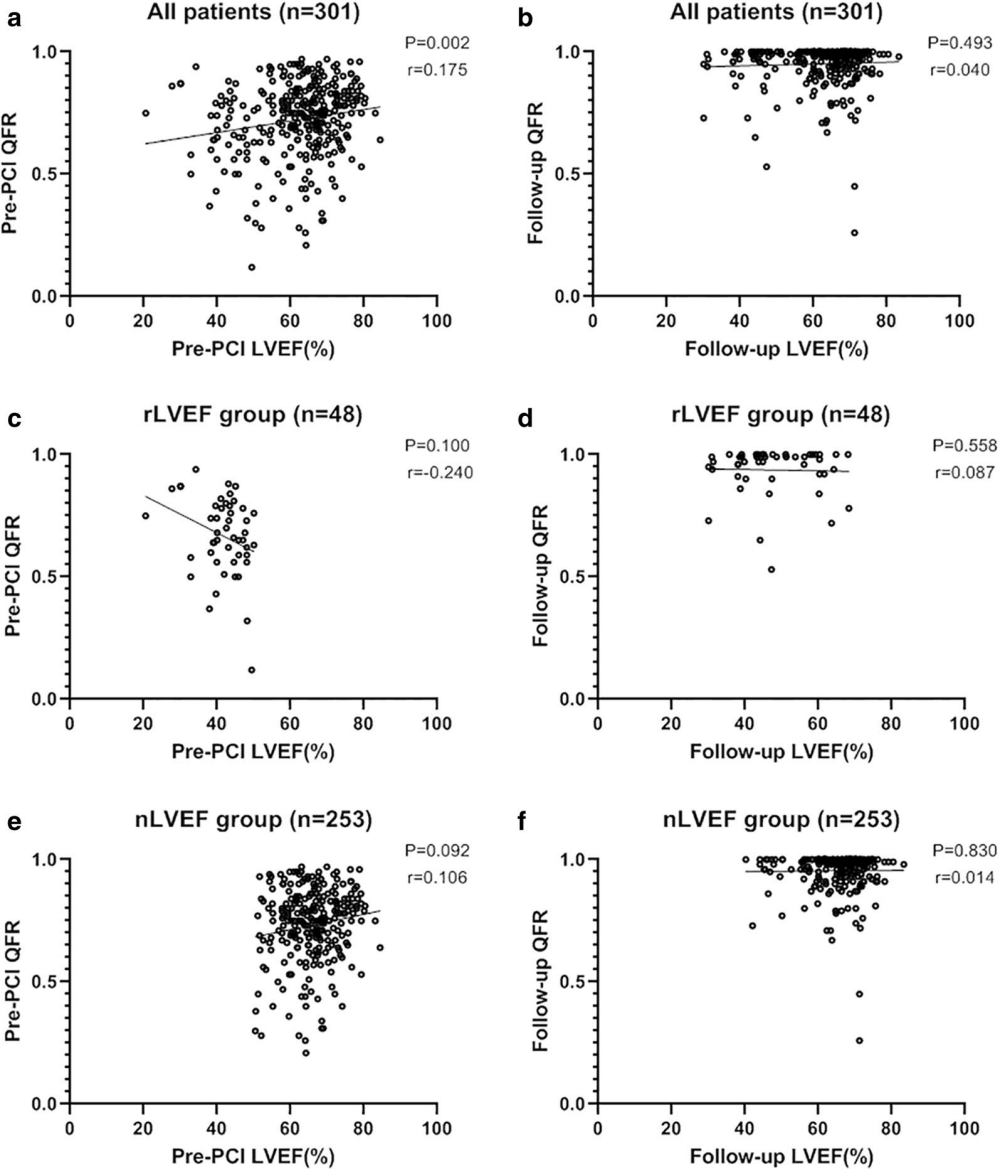
Correlation between QFR and LVEF. QFR, quantitative flow ratio; LVEF, left ventricular ejection fraction. **a**, **b** All patients; **c**, **d** Reduced LVEF group (rLVEF); **e**, **f** Normal LVEF group (nLVEF)

We show the computation results of the pre-PCI, post-PCI and follow-up QFRs in Table 2. Although the pre-PCI QFR value was markedly lower, the rLVEF group had similar post-PCI and follow-up QFR results compared with the nLVEF group (0.96 ± 0.06 vs. 0.97 ± 0.06, *p* = 0.239; 0.94 ± 0.10 vs. 0.96 ± 0.08, *p* = 0.216). The pre-PCI LVEF was not associated with the pre-PCI QFR, and the follow-up LVEF was not related to the follow-up QFR (Fig. 2a, b). When the correlation was analysed individually among subgroups (rLVEF and nLVEF), LVEF was still not significantly associated with QFR (Fig. 2c–f).

**Table 2:** Functional assessments

	Reduced LVEF group (n = 48)	Normal LVEF group (n = 253)	*P* value
Pre-PCI QFR	0.67 ± 0.16	0.73 ± 0.15	0.004
Post-PCI QFR	0.96 ± 0.06	0.97 ± 0.06	0.239
Follow-up QFR	0.94 ± 0.10	0.96 ± 0.08	0.216
Increasing QFR*	0.27 ± 0.18	0.22 ± 0.15	0.043
Post-PCI MLD	3.75 (2.40)	3.40 (2.10)	0.225
Follow-up MLD	2.80 (1.90)	2.80 (1.80)	0.717
LLL	0.60 (1.85)	0.40 (1.70)	0.229
Pre-PCI LVEF, %	41.70 ± 6.21	65.95 ± 7.10	< 0.001
Follow-up LVEF, %	47.39 ± 10.62	65.51 ± 7.36	< 0.001
Increasing LVEF*, %	6.05 ± 9.45	− 0.37 ± 8.11	< 0.001

Values are the mean ± standard deviation, median (interquartile range)

LVEF, left ventricular ejection fraction; PCI, percutaneous coronary intervention; QFR, quantitative flow ratio. MLD, minimal lumen diameter; LLL, late lumen loss

*Increasing QFR = Follow-up QFR – Pre-PCI QFR; Increasing LVEF = Follow-up LVEF – Pre-PCI LVEF

### Variation trend of LVEF and QFR

We computed increasing trends in QFR and LVEF between pre-PCI and follow-up. The results are presented in Table 2. Both groups showed an obvious improvement in QFR, while the rLVEF group showed a higher elevation (0.27 ± 0.18 vs. 0.22 ± 0.15, *p* = 0.043). The rLVEF group showed a significant improvement in LVEF, while the nLVEF group showed slight changes. However, the LVEF in the rLVEF group was still lower (47.39 ± 10.62% vs. 65.51 ± 7.36%, *p* < 0.001). Nonetheless, the LVEF of the rLVEF group showed better improvement than that of the nLVEF group (6.05 ± 9.45% vs. − 0.37 ± 8.11%, *p* < 0.001). In addition, the comparison of MLD and LLL between groups is shown in Table 2. We found similar MLD and LLL results between the two groups, indicating a similar level of functional restenosis between groups.

### Comparisons of clinical outcomes

Comparisons of clinical outcomes at one year between the reduced and normal LVEF patients are demonstrated in Table 3. Despite having a favourable decrease in QFR, the rLVEF group still had a higher incidence of MACCEs (14.6 vs. 4.3%, *p* = 0.016). The rLVEF group had a higher risk of heart failure (6.3% vs. 0%, *p* = 0.004), was is the main reason for the discrepancy in MACCE incidence between the two groups. Nevertheless, the incidences of MI, stroke, and repeat revascularization showed similar results between the two groups. In addition, the occurrence of unplanned cardiac admission was not significantly different between the two groups. Moreover, the improvements in glucose and CRP levels are shown in Table 4. Both groups achieved favourable management of glucose and inflammation. No significant difference in the decrease in glucose levels was reported.

**Table 3:** Comparison of clinical outcomes at one year

	Reduced LVEF group (n = 48)	Normal LVEF group (n = 253)	*P* value
MACCE, n (%)	7 (14.6%)	11 (4.3%)	0.016
Myocardial infarction, n (%)	1 (2.1%)	1 (0.4%)	0.294
Revascularization, n (%)	4 (8.3%)	11 (4.3%)	0.637
Rehospitalization due to heart failure, n (%)	3 (6.3%)	0 (0%)	0.004
Stroke, n (%)	1 (2.1%)	0 (0%)	0.159
Unplanned cardiac admission, n (%)	3 (6.3%)	9 (3.6%)	0.637

Values are n (%)

LVEF, left ventricular ejection fraction; MACCE, major adverse cardiovascular and cerebrovascular event

**Table 4:** Variation in glucose levels and inflammatory status

	Reduced LVEF group (n = 48)	Normal LVEF group (n = 253)	*P* value
*Pre-PCI*			
Diabetes, n (%)	24 (50.0%)	77 (30.4%)	0.008
Prediabetes, n (%)	0 (0.00%)	7 (2.8%)	0.520
Normoglycaemic, n (%)	24 (50.0%)	169 (66.8%)	0.026
Glucose level at admission, mmol/L	6.70 (3.14)	5.54 (1.64)	0.001
CRP, mg/L	5.23 (8.94)	1.69 (4.51)	< 0.001
*Follow-up*			
Diabetes, n (%)	24 (50.0%)	77 (30.4%)	0.008
Prediabetes, n (%)	1 (2.1%)	10 (4.0%)	0.831
Normoglycaemic, n (%)	23 (47.9%)	166 (65.6%)	0.020
Glucose level at follow-up, mmol/L	6.12 (3.04)	5.42 (1.36)	0.001
Follow-up CRP, mg/L	1.37 (3.10)	0.97 (2.49)	0.012
Decreasing glucose level	− 0.12 (2.59)	− 0.05 (1.42)	0.944

Values are mean ± standard deviation, medians (interquartile range), or n (%)

LVEF, left ventricular ejection fraction; PCI, percutaneous coronary intervention; CRP, C-reactive protein

## Discussion

The main findings were as follows: first, one year after PCI, patients with reduced LVEF may show a more increased QFR than patients with normal LVEF, indicating that patients with reduced LVEF achieved better improvement in coronary function from the perspective of flow physiology; second, within the short-term follow-up, CAD patients with reduced LVEF had a similar incidence of restenosis as evaluated by functional assessments and had similar incidences of MI, stroke, and repeat revascularization. However, patients with reduced LVEF still had a higher occurrence of MACCEs, mainly due to a higher incidence rate of heart failure. The diagnostic performance of QFR has been confirmed to be noteworthy in evaluating ischaemia by previous studies [7–9]. Moreover, the importance that cardiologists attach to the prognostic value of QFR is being increasingly considered [11, 12]. The vascular information provided by QFR has been a significant part of flow physiology evaluations. However, research concerning QFR evaluations of the physiological outcomes of PCI in patients with different conditions remains insufficient. Therefore, the findings of our study may enrich the multidimensional assessment of CAD patients and help select the optimal strategy for clinical practice.

In CAD patients with moderate stenosis, the impact of reduced LVEF to QFR measurements is negligible. According to Spearman's correlation analysis, no association was shown between LVEF and QFR at pre-PCI or follow-up. The QFR computation theory may explain this result. QFR has a similar computational formula to FFR. The definition of FFR is (Pd − Pv)/(Pa − Pv), which is simplified to Pd/Pa because Pv is typically ignored in patients with normal LVEF (where Pa = mean proximal coronary pressure, Pd = mean distal coronary pressure, and Pv = mean central venous pressure). FFR may be overestimated for patients with elevated Pv caused by impaired cardiac function, but this overestimation only makes sense when the vessel has severe diameter stenosis (> 90%) [13]. The derivation of QFR is also Pd/Pa [14], which indicates that the significant overestimation caused by a reduced LVEF may occur only when narrow coronary stenosis is present. Our study only included patients with moderate stenosis (50–90%), and the results of the correlation analysis confirmed this hypothesis. Therefore, the results concerning flow physiology in our study were not affected by bias in QFR measurement.

CAD patients with reduced LVEF seem to have a higher incidence of MI and are reported to have lower pre-PCI QFRs. More severe ischaemia and inflammation occur in MI patients, which makes them likely to be stratified into the rLVEF group and show lower QFRs and higher levels of NT-proBNP, CRP, and troponin I simultaneously. Because of an increased risk in heart failure, diabetic patients tend to have poor LVEF [15]. However, no significant difference in the incidence with coexisting prediabetes was found between groups. Notably, altered glucose homeostasis and inflammatory statuses might affect the decrease in the pre-PCI QFR value. Regardless of the degree of coronary artery stenosis, hyperglycaemia is associated with a higher level of nitrotyrosine and is thought to be a marker of oxidative stress. Inflammation and hyperglycaemia both lead to endothelial dysfunction that mainly results from impaired epicardial endothelial-dependent vasodilation [16–19]. Endothelial dysfunction changes coronary haemodynamics and is represented by a decline in QFR. However, a low pre-PCI QFR can be affected by various factors, and the most influential factor is still considered the degree of stenosis.

The patients with reduced LVEF appear to achieve more improvements after PCI based on the cardiac functional assessment. QFR can provide physiological information concerning coronary arteries, while LVEF reflects cardiac systolic function, both of which are important parts of the cardiac functional assessment [2, 7]. Both groups obtained an evident improvement in QFR; furthermore, the elevation in QFR in the rLVEF group was more obvious. Furthermore, only the rLVEF group showed an improved LVEF. This difference indicates more improvements in the functional assessment of the rLVEF group. The correction of coronary microcirculatory dysfunction (CMD) may account for this result. CMD was found to be related to both FFR and LVEF by previous studies [20, 21]. As QFR is an alternative approach for estimating FFR, QFR may also be affected by CMD. When CMD occurs, it induces ischaemia and affects cardiac contractility, resulting in poor QFR and LVEF values. After revascularization, these microvascular disorders are gradually corrected, which is reflected in the improvements in QFR and LVEF. With a higher incidence of CMD, rLVEF patients gain more physiological benefits from CMD correction. The basic satisfactory LVEF level also limited the elevation in LVEF in the nLVEF group. In addition, we found that the levels of glucose and CRP improved well with the use of hypoglycaemic therapy. A previous study demonstrated that hypoglycaemic drugs may directly ameliorate the degree of stenosis [22]; as a result, the increase in QFR may be associated with decreases in glucose and CRP. However, the decreases in glucose level showed were a similar between the two groups, indicating that glucose level was not the main factor contributing to the improvement in QFR.

Concerning the clinical outcomes, we found that the rLVEF group had a higher incidence of MACCEs, which was mainly caused by a higher incidence of heart failure. Consisting of patients with poor cardiac function, the rLVEF group was expected to have a higher risk of heart failure. However, rLVEF patients had a similar incidence of MACCEs, regardless of the incidence of heart failure and better improvements in physiological assessment. Compared to nLVEF patients, rLVEF patients who underwent successful PCI seemed to show better improvement based on functional assessments and analogous short-term outcomes with respect to MI, stroke, and revascularization. In addition, restenosis is associated with altered endothelial function [23]. Therefore, we also assessed the degree of restenosis from the view of flow physiology. LLL is an index to evaluate the absolute degree of restenosis and the status of intimal hyperplasia in the coronary artery [24]. There were similar LLL results between the two groups, suggesting a similar degree of functional restenosis. Although they had a worse status in the pre-PCI period, patients with reduced LVEF did not show a worse clinical outcome compared to patients with normal LVEF in the short-term follow-up. It seems that more physiological benefits from successful revascularization were achieved by patients with reduced LVEF.

It remains controversial whether revascularization by PCI leads to more benefits in patients with reduced LVEF than medical treatment [25, 26]. Although previous studies and meta-analyses have shown the benefits of PCI in improving survival for patients with CAD and reduced LVEF [27, 28], some cardiologists still face a conundrum in choosing revascularization for this group of patients because of the lack of a proper evaluation method. Therefore, our study provides new evidence based on QFR to support the use of the revascularization strategy in patients with reduced LVEF from the perspective of flow physiology. QFR may have great potential in the assessment of CAD patients in the future.

This study still has some limitations. Our study is a retrospective single-centre observational study with a small sample size. The results of this study need to be further verified by prospective multicentre cohort studies in the future. In addition, not all images are suitable for QFR analysis, and this consideration may affect the selection process of patients during QFR measurement. Additionally, the measurement of QFR may be affected by CMD [29]. As our study is a pilot study, the influence of glucose level and inflammation on QFR still needs further exploration.

## Conclusion

After successful PCI, patients with reduced LVEF were reported to have greater increases in QFR and LVEF than patients with normal LVEF; furthermore, patients with reduced LVEF had a similar degree of restenosis and a higher incidence of MACCEs due to a higher risk of heart failure. It seems that this group of patients gains more coronary benefits from successful revascularization from the perspective of flow physiology evaluations.

## Data Availability

The datasets generated and/or analysed during the current study are not publicly available due to privacy or ethical restrictions, but are available from the corresponding author on reasonable request.

## Abbreviations

AMI: Acute myocardial infarction
CAD: Coronary artery disease
CMD: Coronary microcirculatory dysfunction
CRP: C reaction protein
DES: Drug eluting stent
FFR: Fractional flow reserve
LVEF: Left ventricular ejection fraction
LDL: Low density lipoprotein
LLL: Late lumen loss
MACCE: Major adverse cardiovascular and cerebrovascular events
MLD: Minimal lumen diameter
NT-proBNP: N-terminal pro brain natriuretic peptide
PCI: Percutaneous coronary intervention
QFR: Quantitative flow ratio
TIMI: Thrombolysis in myocardial infarction
